# Therapeutic effect of subcutaneous injection of low dose recombinant human granulocyte-macrophage colony-stimulating factor on pulmonary alveolar proteinosis

**DOI:** 10.1186/s12931-019-1261-1

**Published:** 2020-01-02

**Authors:** Fen Zhang, Dong Weng, Yiliang Su, Chengsheng Yin, Li Shen, Yuan Zhang, Ying Zhou, Qiuhong Li, Yang Hu, Huiping Li

**Affiliations:** Department of Respiratory Medicine, Shanghai Pulmonary Hospital, Tongji University, School of Medicine, 507 Zheng Min Road, Shanghai, 200433 China

**Keywords:** Pulmonary alveolar proteinosis, Recombinant human granulocyte-macrophage colony-stimulating factor, Injection

## Abstract

**Objective:**

To observe the efficacy of recombinant human granulocyte-macrophage colony-stimulating factor (rhGM-CSF) for pulmonary alveolar proteinosis (PAP).

**Materials and methods:**

A total of 55 patients with PAP were screened at Shanghai Pulmonary Hospital between May 2014 and May 2018. Among these, 42 were diagnosed with idiopathic PAP, 24 were included in this study, 20 were treated for 6 months, and 17 were followed up for additional 6 months. All patients received a subcutaneous injection of 75μg/d GM-CSF qd for 1 month. The therapeutic dose was adjusted according to the changes in the lesions of chest CT. If the lesions were absorbed, subcutaneous injections of 75μg/d GM- CSF qd and 75μg/d GM-CSF qod were given for 2 and 3 months, otherwise, the dose was increased to 150μg/d GM-CSF qd and 150μg/d qod for 2 and 3 months, respectively. All cases were treated once a day in the first 3 months and once every other day in the last 3 months. The total course of treatment was 6 months. After withdrawal, the patients were followed up for another 6 months. The deadline of follow up was September 30, 2019.

**Results:**

Twenty patients completed the treatment and efficacy evaluation. One patient was completely cured, 16 cases improved, three cases were noneffective. After 1-month evaluation, 12 patients received an increased dose (150μg) from the second month of treatment. Seventeen patients completed the 12-month follow-up, among which fourteen improved. CT showed the lesions were slightly increased in three cases. Economic burden was the following: RMB 7324–15,190 Yuan were required for the 6-month treatment course, which is significantly lower compared to other treatment methods.

**Conclusion:**

Subcutaneous injection of rhGM-CSF at low dose (75μg-150μg /d) is effective treatment for patients with idiopathic PAP.

**Trial registration:**

NCT01983657. Registered 16 April 2013.

## Background

Pulmonary alveolar proteinosis (PAP) is a rare, diffuse lung disease of unknown etiology, characterized by the filling of the alveolar cavity with phospholipid and protein-containing periodic acid- schiff (PAS) staining positive granular substances. PAP was discovered in 1958 by three pathologists, SH Rosen, Benjamin Castleman and AA Liebow, thus is also known as Rosen-Castleman-Liebow’s Triad Syndrome [1].

PAP consists of three types: congenital, secondary and idiopathic (also known as acquired or autoimmune) [2–4]. Idiopathic PAP is the main type of PAP, accounting for more than 90%, and is associated with autoimmune abnormalities [5, 6]. GM-CSF is a hematopoietic cell growth-stimulating factor that not only stimulates the proliferation and differentiation of bone marrow cells and their stem cells, but also regulates the phagocytic function of alveolar macrophages and their ability to degrade surfactants, having an important role in the balance of pulmonary surfactants. In 1994, Dranoff et al [7] have found an association between pulmonary alveolar proteinosis and GM-CSF; pulmonary alveolar proteinosis was found in mice with GM-CSF-encoding gene deletion, while the expression of GM-CSF in lung tissue by transgenic technology could make protein-like substances of alveolar deposition in gene knockout mice disappear. Its pulmonary pathological changes could be restored to normal through re-expression of GM-CSF in the lung tissue. Nevertheless, no deletion or abnormalities of GM-CSF coding gene have been found in patients with PAP [8]. Moreover, a number of studies [9–13] have indicated that autoantibodies are able to neutralize granulocyte-macrophage colony-stimulating factor in alveolar lavage fluid or serum samples from patients with idiopathic PAP; however, this effect was not found in secondary PAP, healthy controls and other patients with lung disease. The presence of this antibody impairs the function of granulocyte-macrophage colony-stimulating factor, resulting in increased number of alveolar lipoproteins that eventually lead to pulmonary alveolar proteinosis. Takaki and colleagues [13] have found that patients with pulmonary alveolar proteinosis relapse after double lung transplantation, further confirming the view that “the presence of autoantibodies neutralizes neutrophil-macrophage colony-stimulating factor in patients with idiopathic PAP”. Subsequently, some studies have extracted anti-GM-CSF antibodies from the blood of PAP patients and used it in healthy non-human primates, proving that the biochemical, cytological and pathological characteristics of pulmonary alveolar proteinosis could be observed via this approach [14]. Based on these studies, it is believed that the presence of anti-GM-CSF antibodies in the body is an important cause of idiopathic pulmonary alveolar proteinosis. Therefore, the ability of alveolar macrophages to degrade surfactants should be partially restored by supplementing GM-CSF, thus achieving the purpose of treating PAP.

Thus far, many studies have reported the treatment of PAP with GM-CSF. However, no uniform standard existed. The main methods followed were inhalation therapy and subcutaneous therapy. The time of therapy included daily medication and different time intervals. The total course of treatment varied from 3 to 36 months, and the doses were different between studies [15–24] (Table 1). The dose of subcutaneous injection was from 3 to 20 μg/(kg · day) and the time course of the therapy was from 3 to 68 weeks, which brought huge financial burden and side effects. The minimum dose of inhalation therapy was 125 μg qd [22, 24], and the maximum dose was 1000 μg/day [21]. The use of atomizing device also brought the risk of secondary infection to patients. Although the efficacy was positive, these treatment approaches had some drawbacks, such as high dose, high cost, and increased risk of infection due to atomized devices. The clinical experience in the present study indicated that some patients with idiopathic PAP responded well to lower doses of GM-CSF (75 μg/day to 150 μg/day) by a subcutaneous injection and had lower financial burden. A clinical trial named “clinical observation of subcutaneous injection of low-dose rhGM–CSF on treatment of PAP” (ID number is NCT01983657) was registered to further verify the efficacy of low-dose recombinant human granulocyte-macrophage colony-stimulating factor (rhGM-CSF) for PAP. The goal of this study was to explore the efficacy and safety of a lower dose of rhGM-CSF for idiopathic PAP, which should also be convenient to perform and help reduce the cost of therapy.

**Table 1:** Literature summary of GM-CSF treatment for PAP

Trials	Usage of GM-CSF	Maximum dose of GM-CSF	Medication time^a^	Effective rate	Year^b^
Seymour et al. [15]	subcutaneous injection	5 μg/kg/day	300 days	100% (*n* = 1)	1996
Kavuru et al. [16]	subcutaneous injection	9μg/kg/day	12 weeks	75% (*n* = 4)	2000
Seymour et al [17]	subcutaneous injection	20μg/kg/d	6–12 weeks	43%(*n* = 14)	2001
Bonfield et al. [18]	subcutaneous injection	18 μg/kg/day	12–48 weeks	55% (*n* = 11)	2002
Tazawa et al. [19]	inhalation therapy	250 μg/day; every other week	24 weeks	100% (*n* = 3)	2005
Venkateshiah et al. [20]	subcutaneous injection	18 μg/kg/day	12–52 weeks	48% (*n* = 21)	2006
Wylam et al. [21]	inhalation therapy	500ugbid week-on, week-off	3–68 weeks	92%(*n* = 12)	2006
Tazawa et al. [22]	inhalation therap	250 μg/day d1-d8, d9-d14 no use of drugs × 6 cycles	24 weeks	62% (*n* = 39)	2010
Spyros et al. [23]	inhalation therapy	250μg qd 4 days-on, 4 days-off	14–65 weeks	100%(*n* = 6)	2014
*Tazawa* et al. [24]	inhalation therapy	(125 μg bid d1-d8, d9-d14 no use of drugs × 6 cycles) + (125 μg qd d1-d4,d5-d14 no use of drugs× 6 cycles)	24 weeks	66%(*n* = 35	2014

*GM-CSF* Granulocyte-macrophage colony-stimulating factor, *PAP* Pulmonary alveolar proteinosis

^a^Medication time:The time of administration was fixed in some studies, while varied in some studies. In this situation, the medication time was showed as the shortest time - the longest time in this table

^b^year: The publication time of the study

## Materials and methods

### Inclusion and exclusion criteria

**Fig. 1:**
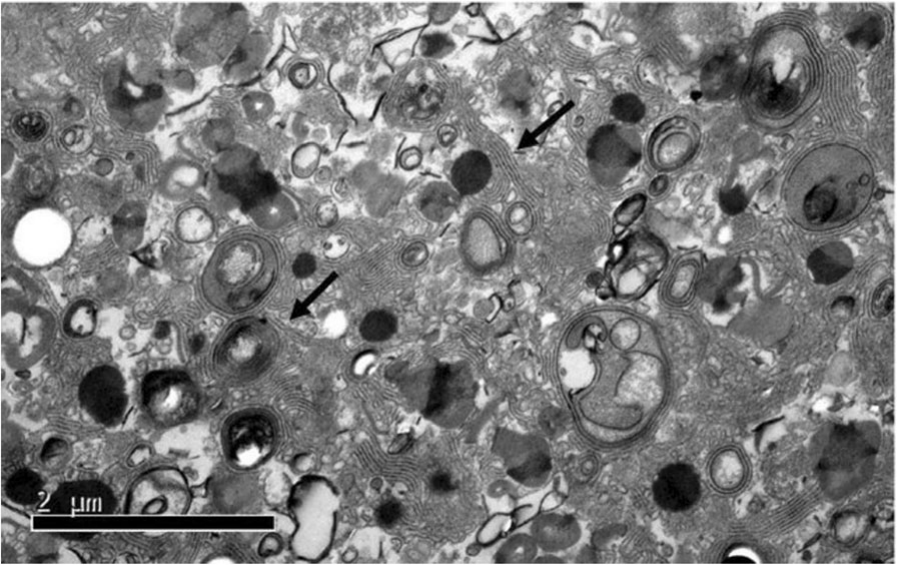
Electron microscopic appearance of patient’s lavage fluid. Transmission electron microscopy (TEM) showing oval granules with bright and dark interlaced threadlike or fingerprint-like stripes. × 20,000 times

**Fig. 2:**
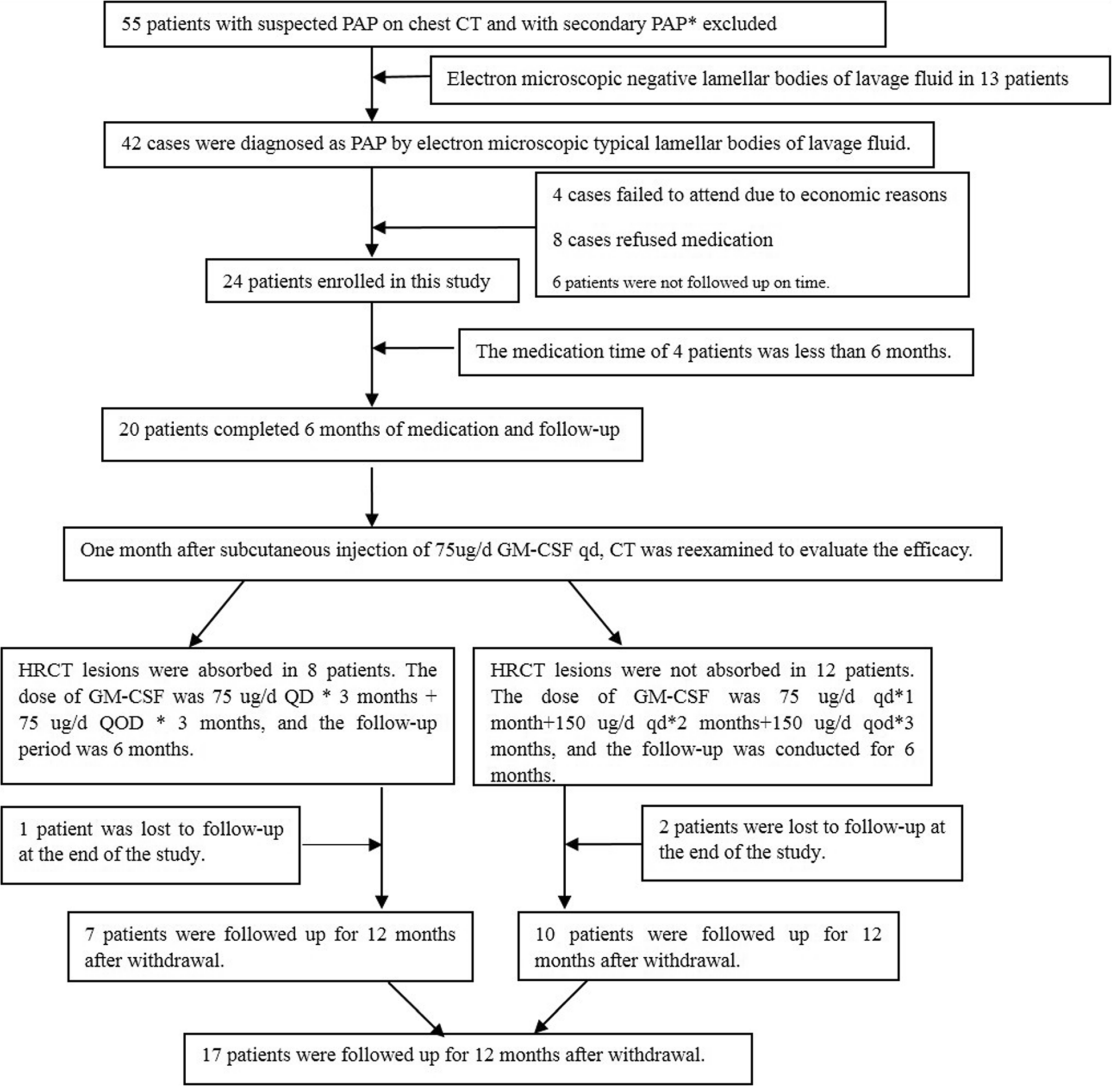
Patient screening process, * Methods to exclude secondary PAP: Medical history was inquired to exclude genetic factors; to inquire whether there is a history of industrial dust exposure and ask pneumonologists to help exclude occupational pneumoconiosis; Microbial examination was performed to exclude secondary PAP changes caused by viruses, bacteria, fungi, tuberculosis and other infections through sputum, bronchoalveolar lavage fluid, blood and other samples

A total of 55 patients with PAP who were admitted at the Shanghai Lung Hospital from May 2014 to May 2018 were included in this study. Diagnostic criteria for PAP [25–27] were the following: 1) patients with or without cough and polypnea; 2) chest HRCT showing typical paving stone signs; 3) under electron microscope, oval granules were observed in the lavage solution, with bright and dark threaded or fingerprint-like stripes, which suggested the presence of lamellar bodies (Fig. 1); 4) secondary factors such as industrial dust exposure, various infectious diseases, malignant hematological diseases or immunodeficiency diseases were excluded. The inclusion criteria were: (1) diagnosis of idiopathic pulmonary alveolar proteinosis according to PAP diagnostic criteria; (2) 18–70 years old, both male and female; (3) Informed consent. Exclusion criteria were the following: (1) patients with secondary pulmonary alveolar proteinosis; (2) patients who received rhGM-CSF treatment within 6 months before enrollment; (3) those who received whole lung lavage treatment within 4 weeks before enrollment; (4) leukocyte count (> 12,000/ul); (5) fever > 38 °C; (6) severe edema: systemic diseases, such as cardiac insufficiency, liver and kidney insufficiency, etc.; (7) patients with severe diseases of other systems, including liver, kidney, lung, blood and cardiovascular diseases; (8) pregnant women or those planning the pregnancy, and lactating women; (9) patients with severe history of drug allergy or allergy constitution, or those allergic to *Escherichia coli* preparations; (10) unable to express subjective discomfort symptoms; (11) patients who are allergic to GM-CSF or not suitable for GM-CSF; (12) other conditions determined by the investigator that do not meet the inclusion criteria. Finally, 42 patients were diagnosed with PAP, 24 patients were included in the study. Twenty patients completed 6 months treatment and 6 months follow-up; 17 patients completed 12 months follow-up (Fig. 2), including 3 female patients and 17 male patients.

### Drug administration and evaluation scheme

**Fig. 3:**
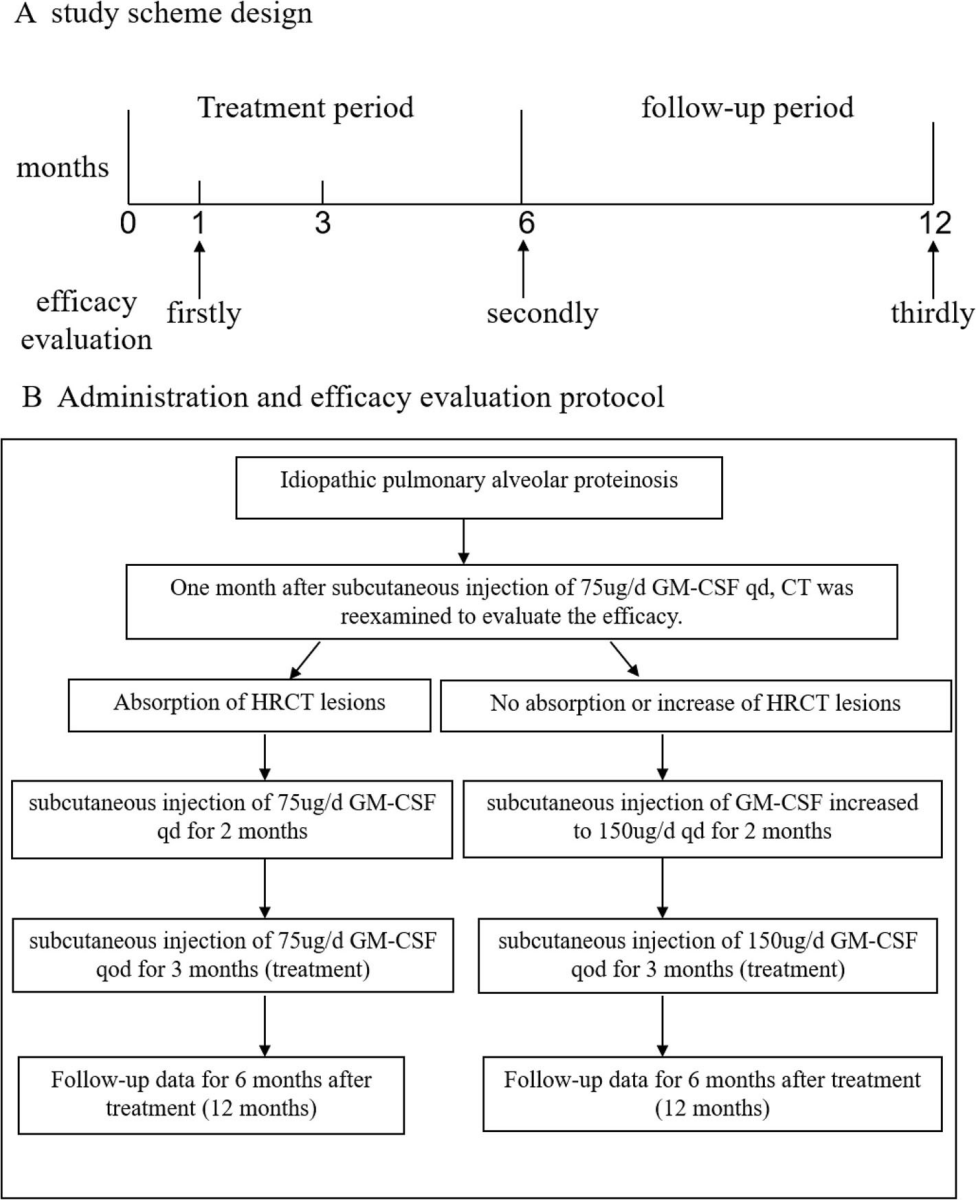
**a** Study Scheme Design. **b** Administration and efficacy evaluation protocol

The drug administration and evaluation scheme are presented in Fig. 3. Briefly, enrolled patients were given subcutaneous injection of 75μg/d GM-CSF qd for 1 month. Consequently, all patients underwent chest CT. According to the observation of preclinical medication, the efficacy for 3 months has been relatively stable. Therefore, this study was designed as follows: once a day for the first 3 months and once every other day for the next 3 months after the evaluation of the efficacy. We hope to achieve the purpose of not only maintaining the treatment effect but also reducing the economic burden. The results of this study also confirm the rationality of this study design. If the lesions were absorbed, subcutaneous injection of 75μg/d GM-CSF qd for 2 months and subcutaneous injection of 75μg/d GM-CSF qod for 3 months were continued. After 1 month, if the chest CT showed no absorption or increase, the dose was increased to 150 μg/d GM-CSF qd for 2 months and 150 μg/d GM-CSF qod for 3 months. After that, all patients were followed up to 12 months.

### Evaluation of efficacy and safety

#### Evaluation indexes of efficacy

Treatment efficacy was calculated based on chest CT paving stone sign proportion score (Table 2), lung function (FVC, DLCO%), blood gas analysis (oxygen partial pressure, alveolar-arterial oxygen partial pressure difference), six-minute walking test, symptom score (score of cough and polypnea) (Table 3).

**Table 2:** Imaging examinations (scoring method according to the proportion of paving stone signs displayed on CT)

□	0 point	no paving stone signs
□	1 point	paving stone signs less than 5%
□	2 points	5–24% of paving stone signs
□	3 points	25–49% of paving stone signs
□	4 points	50–74% of paving stone signs
□	5 points	paving stone signs more than≥75%

**Table 3:** Clinical symptom score

Polypnea Or dyspnea	□1 point Dyspnea only when exerting themselves.
	□2 points Shortness of breath when walking fast on flat ground or climbing small slopes on foot.
	□3 points Because of shortness of breath, walking on flat ground is slower than that of their peers or requires stopping to rest.
	□4 points Stop to catch breath after walking on flat ground for about 100 m or a few minutes
	□5 points Severe dyspnea prevents the patient from leaving his home or dyspnea while dressing or undressing.
cough	□0 point No cough during the day; no cough at night.
	□1 point There are occasional short coughs in the daytime; temporary coughs or occasional coughs when sleeping at night.
	□2 points Frequent cough during the day slightly affects daily activities; cough slightly affects sleep at night.
	□3 points Frequent cough during the day seriously affects daily activities; cough seriously affects sleep at night.
	Other clinical symptoms:

#### Efficacy evaluation

Curative effect was calculated based on clinical symptoms and signs, imaging data, arterial blood gas, pulmonary function, etc.. According to the study protocol, all patients included in the analysis were evaluated three times at 1 month of treatment, completion of 6-month medication (6 months) and 6 months of follow-up (12 months) after withdrawal. Three grades were used: (1) Cure: all clinical symptoms and abnormal signs disappeared; arterial blood gas and pulmonary function returned to normal levels and no abnormalities were visible on chest CT scan; (2) Improvement: chest CT showed improved images, and pulmonary function, arterial blood gas, clinical symptoms and signs all improved; (3) inefficacy or deterioration: no improvement or deterioration of imaging, pulmonary function, arterial blood gas, clinical symptoms and signs were observed. Cases with cure and improvement were effective ones.

#### Evaluation indexes of safety

Evaluation indexes of safety were based on blood routine, liver function, renal function, electrolyte, myocardial zymogram, electrocardiogram; and eventual adverse events.

### Drug cost estimation

According to the therapeutic regimen and the current drug market price, the costs of each study treatment were calculated based on the following formula: *time of administration × the dose of the drug × the unit price of the drug*. In addition, the minimum cost (the shortest time × the minimum dose × the unit price of the drug) and the maximum cost (the longest time × the maximum dose × the unit price of the drug) were also calculated.

### Statistical analysis

Related data of all eligible PAP patients were statistically analyzed. Pulmonary function (FVC, DLCO%), blood gas analysis (oxygen partial pressure, alveolar-arterial oxygen partial pressure difference), six-minute walking distance and other continuous numerical variables were analyzed by paired sample t test. The specific values were recorded by mean ± standard deviation. *P* < 0.05 was considered to be statistically significant. Orderly categorical variables such as Chest CT paving stone sign proportion score, symptom score (score of cough and polypnea) were analyzed by Wilcoxon symbolic rank test. Results of efficacy analysis were compared and described using percentages. During the clinical study, all patients were followed up to record the frequency, time and severity of adverse events, and the safety analysis was descriptive.

## Results

Twenty patients completed 6 months of treatment, including 17 males and 3 females (average age of 46.65 ± 10.84 years old). Seventeen patients were followed up for 6 months after the treatment was completed.

### Evaluation of efficacy after treatment (6 months)

#### Chest CT data

**Fig. 4:**
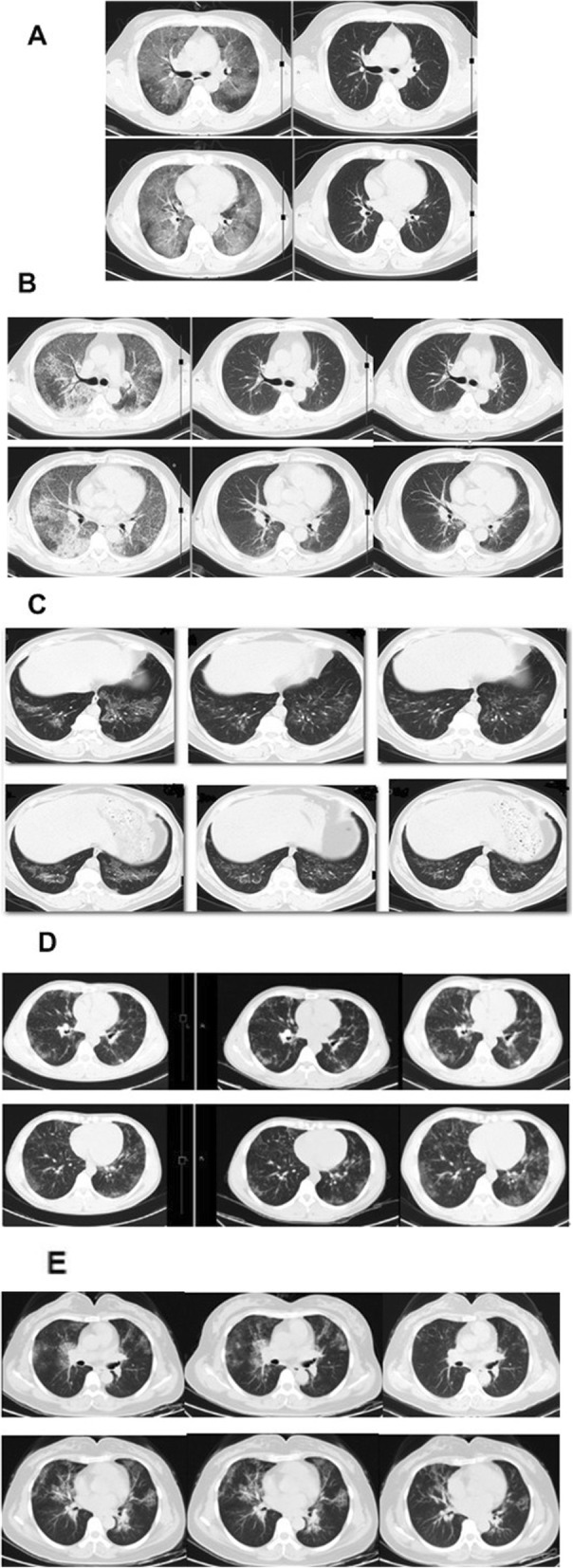
Changes of chest CT before and after treatment, **a** CT contrast of complete absorption of the lesion after 6 months of treatment. After 6 months of treatment, the lesion was completely absorbed. **b** CT contrast of apparent absorption of the lesion after treatment. The re-examination of chest CT showed that the lesion was obviously absorbed after 6 months of treatment. After 6 months of treatment, the patient stopped taking medicine and were followed up for additional 6 months (12 months); further slow absorption of the lesion was shown on chest CT. **c** CT contrast of partial absorption of the lesion after treatment. The re-examination of chest CT showed slight absorption after 6 months of treatment. The patient stopped taking medicine after 6 months of treatment. Follow-up of chest CT at 12 months showed that the lesion was stable. **d** Slight absorption of the lesion after 6 months of treatment and slight increase of the lesion after withdrawal for 6 months. The re-examination of chest CT showed similar lesions after 6 months of treatment. After the completion of 6 months of treatment, the patient stopped taking medicine, and the follow-up chest CT showed slightly increased lesions at 12 months. **e** The treatment of GM-CSF 75μg /d for 1 month showed poor efficacy, and the lesion was absorbed after the dose was increased to 150μg/d. The patient received GM-CSF 75μg /d treatment for 1 month, and the re-examination of chest CT showed no absorption, so the amount was increased to 150μg/d for 1 month, and the re-examination of chest CT showed absorption

Chest CT examination indicated complete absorption of the lesion in one patient, partial absorption in 16 patients, and no changes in 3 patients (Fig. 4). The paving stone sign proportion scores before and after the treatment were compared with Wilcoxon symbolic rank test, and the results showed that the lesions were absorbed (z = 3.686, *P* = 0.000003; Table 5a).

#### Arterial blood gas

**Fig. 5:**
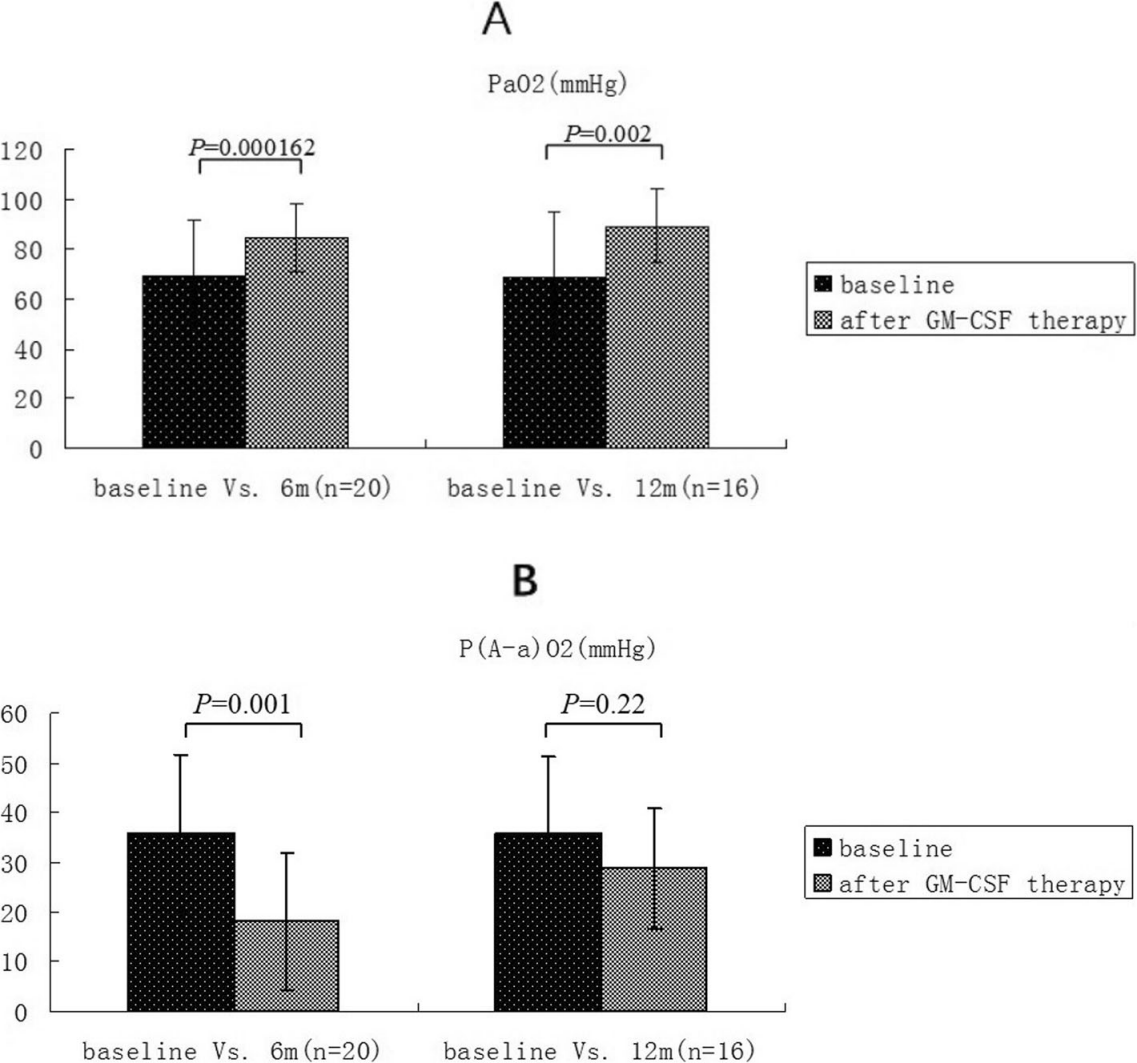
Blood gas analysis before and after treatment: contrast of oxygen partial pressure, **a***, alveolar-arterial oxygen partial pressure difference* (**b**)*. The oxygen partial pressure in arterial blood gas* (**a**) was improved before and after 6 months of treatment, and the difference was statistically significant. Seventeen patients were followed up after 12 months and 16 patients completed blood gas analysis, the oxygen partial pressure at 12 months was also improved compared with that before treatment, and the difference was statistically significant. Alveolar-arterial oxygen partial pressure difference (**b**) was also improved before and after 6 months of treatment, and the difference was statistically significant. Seventeen patients were followed up after 12 months and 16 patients completed blood gas analysis, the difference of alveolar-arterial oxygen partial pressure at 12 months was also improved compared with that before treatment, with no significant difference

The mean ± standard deviation of oxygen partial pressure (Table 4, Fig. 5a) before and after treatment were 69.05 ± 11.14 and 88.16 ± 15 respectively (*p* = 0.000162) while the mean ± standard deviation of alveolar-arterial oxygen partial pressure (Table 4, Fig. 5b) before and after treatment were 35.77 ± 16 and 18.16 ± 13.69 respectively (*p* = 0.001).

**Table 4:** General information of patients and statistical summary of continuous numerical observation indexes

Indexes	Before treatment VS 6 months (*n* = 20)			Before treatment VS 12 months (*n* = 17)		
	Before treatment	6 months	*P* value	Before treatment	12 months	*P* value
Oxygen partial pressure(mmHg)	69.05 ± 1.14	88.16 ± 15	< 0.001	67.56 ± 11.42	81.25 ± 13.55	0.002
Alveolar-arterial oxygen partial pressure difference(mmHg)	35.77 ± 16.00	18.1 ± 13.69	0.001	35.84 ± 15.52	28.75 ± 12.21	0.220
FVC(L)	3.34 ± 0.88	3.54 ± 0.83	0.022	3.165 ± 0.952	3.365 ± 0.849	0.139
DLCO (%)	69.65 ± 22.49	84.49 ± 13.73	0.004	68.75 ± 26.02	89.24 ± 14.70	0.010
Six-minute walking distance(miles)	501.9 ± 133.22	645 ± 117.18	0.002	486.0 ± 134.0	624.0 ± 149.7	0.021

*FVC* Forced vital capacity, *DLCO* Carbon monoxide diffusion capacity

Pulmonary function, blood gas analysis and six-minute walking distance test results (mean ± standard deviation)

#### Pulmonary function

**Fig. 6:**
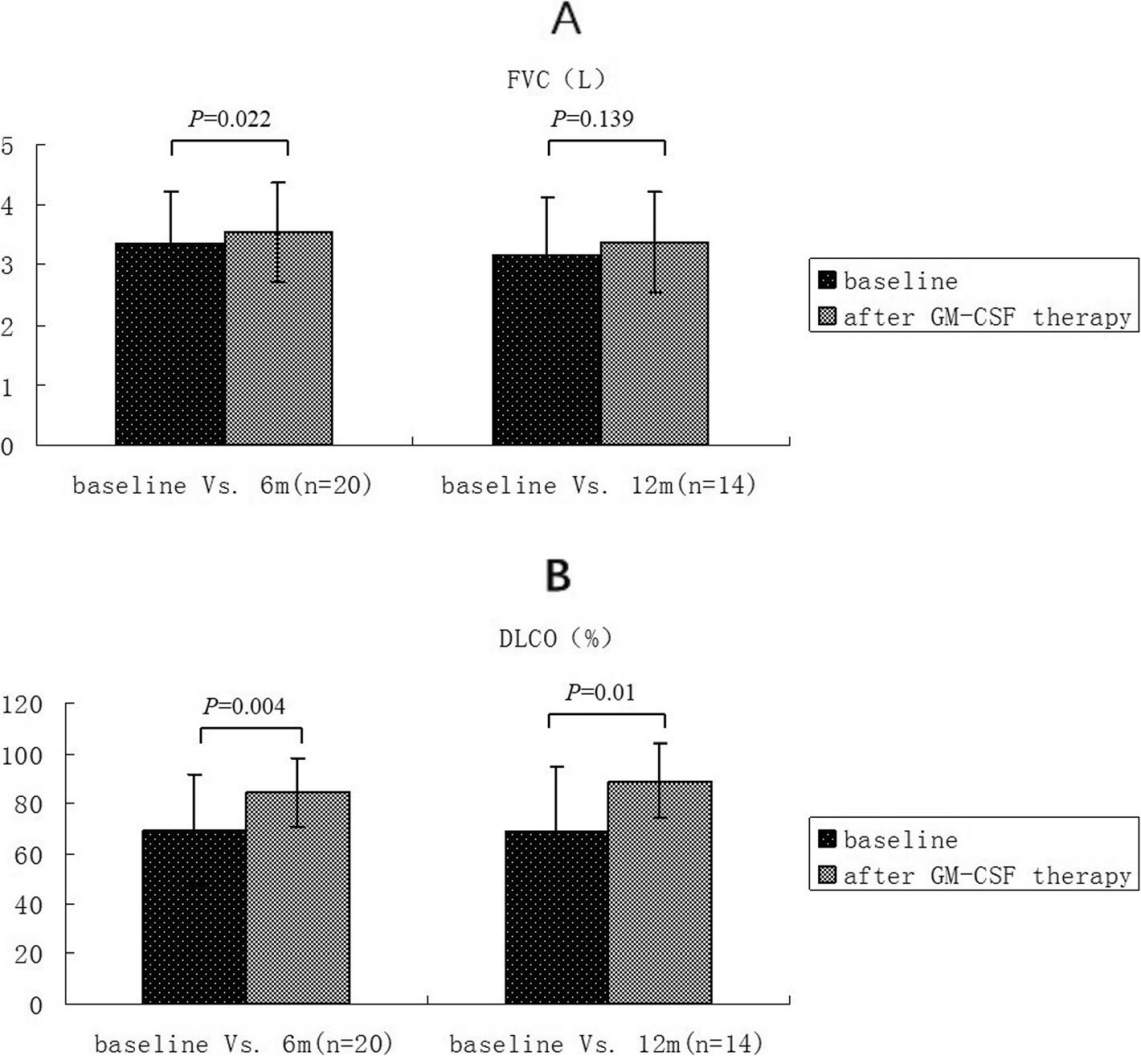
Contrast of pulmonary function indexes (FVC, DLCO%) before and after treatment, FVC (**a**) improved after 6 months of treatment, and the difference was statistically significant. Seventeen patients were followed up after 12 months and 14 patients completed lung function examination, FVC was improved at 12 months compared with that before treatment, and the difference was not statistically significant. DLCO% (**b**) was also improved after treatment for 6 months, and the difference was statistically significant. Seventeen patients were followed up after 12 months, and 14 patients completed lung function examination, DLCO% was also improved at 12 months compared with that before treatment, and the difference was statistically significant

The mean ± standard deviation of FVC (Table 4, Fig. 6a) before and after treatment were 3.34 ± 0.88 and 3.54 ± 0.83, respectively (*p* = 0.022), while the mean ± standard deviation of DLCO% (Table 4, Fig. 6b) before and after treatment were 69.65 ± 22.49 and 84.49 ± 13.73, respectively (*p* = 0.004).

#### Symptom score

The results showed that the cough and polypnea improved after treatment, and the difference was statistically significant (cough: z = 3.419, *P* = 0.001, Table 6; polypnea: z = 3.562, *P* = 0.000011, Table 7).

#### Six-minute walking test

**Fig. 7:**
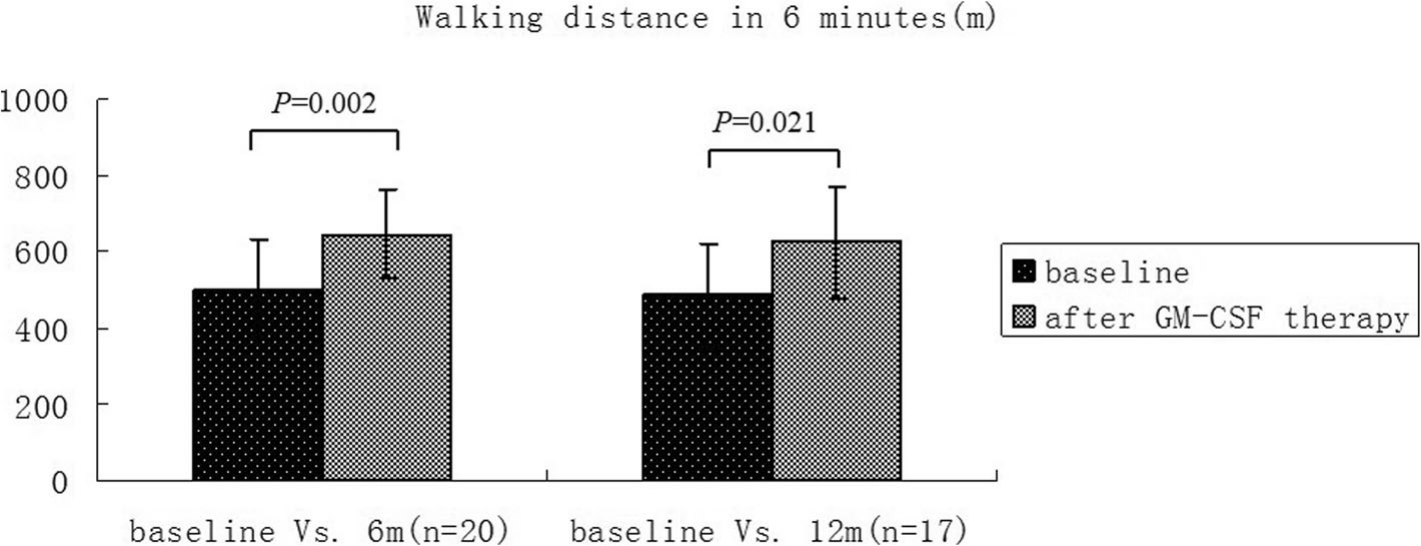
Contrast of six-minute walking distance before and after treatment. After 6 months of treatment, the walking distance of patients was prolonged, and the results were statistically significant. Seventeen patients were followed up after 12 months and the walking distance was also prolonged at 12 months compared with that before treatment, and the difference was statistically significant

Six-minute walking test is shown in Table 4 and Fig. 7. Briefly, 6 months after treatment, the walking distance of patients was prolonged, and the results were statistically significant. The mean ± standard deviation before and after treatment was 501.9 ± 133.2 and 645 ± 117.18, respectively (*p* = 0.002).

#### Judgment of efficacy

Among the 20 patients, 1 patient was cured, 16 patients improved, and 3 patients showed no changes after 6 months treatment, with an improvement rate of 17/20 (85%) (Table 8).

### Evaluation of efficacy after 12 months fellow up

A total of 17 of the 20 patients completed the 12-months follow-up. The comparison of Chest CT paving stone sign proportion scores before and after the treatment (Table 5b) was statistically significant (z = 4.103, *P* = 0.000041). In addition, the comparison of polypnea scores before and after treatment was statistically significant (Table 6b) (z = 3.830, *P* = 0.000128). Moreover, compared with those before treatment, arterial blood gas (Table 4 and Fig. 5), pulmonary function (Table 4 and Fig. 6), cough score (Table 6b) and six-minute walking distance test (Table 4 and Fig. 7) showed improving trend, nevertheless the statistical difference could not be reflected due to small number of cases.

**Table 5:** Comparisons of CT paving stone sign score before and after treatment

(A) Changes of CT paving stone sign score before treatment and after 6 months of treatment (*n* = 20)		
Scores before treatment	Scores after 6 months of treatment	Number of cases
2	1	4
2	2	2
3	1	4
3	2	4
3	3	1
4	1	1
4	2	1
5	0	1
5	1	2
(B) Changes of CT paving stone signs score before treatment and after 12 months of treatment (n = 17)		
Scores before treatment	Scores after 12 months of treatment	Number of cases
2	1	3
2	2	1
3	1	5
3	2	1
3	3	2
4	3	1
4	1	1
5	1	2
5	2	1

Compared with that before treatment, the proportion scores of chest CT paving stone sign improved significantly after 6 months of treatment, with significant difference (z = 3.686, *P* = 0.000003). Seventeen patients were followed up for 6 months (12 months) after treatment, and their CT scores improved significantly compared with that before treatment, with statistical significance (z = 4.103, *P* = 0.00004)

**Table 6:** Comparisons of cough scores before and after treatment

(A) Changes of cough scores before treatment and after 6 months of treatment (*n* = 20)		
Scores before treatment	Scores after 6 months of treatment	Number of cases
0	0	2
1	0	1
1	1	5
2	1	10
3	1	2
(B) Changes of cough scores before treatment and after 12 months of treatment (*n* = 17)		
Scores before treatment	Scores after 12 months of treatment	Number of cases
0	0	1
1	0	1
1	1	3
1	2	1
2	1	8
2	2	1
3	1	2

The cough score was improved before and after 6 months of treatment, with significant difference (z = 3.419, *P* = 0.001); Seventeen patients were followed up after 12 months and the cough after 12 months of treatment improved when compared with that before treatment, with significant difference (z = 2.578, *P* = 0.010)

**Table 7:** Comparison of polypnea scores before and after treatment

(A): Changes of polypnea scores before treatment and after 6 months of treatment (*n* = 20)		
Scores before treatment	Scores after 6 months of treatment	Number of cases
1	1	4
2	1	4
3	1	4
3	2	3
4	1	4
5	1	1
(B): Changes of polypnea scores before treatment and after 12 months of treatment (*n* = 17)		
Scores before treatment	Scores after 12 months of treatment	Number of cases
1	0	1
1	1	2
2	0	1
2	1	1
2	3	1
3	0	2
3	1	3
3	2	1
3	3	1
4	1	3
5	1	1

The score of polypnea was also improved before and after 6 months of treatment, with significant difference (z = 3.562, *P* = 0.000011). Seventeen patients were followed up after 12 months, and compared with that before treatment, the polypnea after 12 months of treatment was also improved, with statistically significant difference (z = 3.830, *P* = 0.000128)

**Table 8:** Evaluation of efficacy

	Cure	Improved	Invalidity or deterioration	Effective cases (Effective rate)
End of treatment(6 months)	1	16	3	17/20(85%)
End of follow-up (12 months)	0	14	3	14/17(82.35%)

**Table 9:** Estimates of funds

Medication method	Minimum Dose x Minimum Time	RMB (yuan)	Maximum Dose x Maximum Time	RMB (yuan)
Inhalation	250μg qd × 14 weeks, 4 days-on, 4 days-off	15,190	500μg bid*68 weeks	344,306
Subcutaneous injection	9μg/kg/day*12 weeks	32,811	18μg/kg/d*52 weeks	284,357
This method	75μg qd*6 months	7324	150μg qd*6 months	13,020

### Cost

Comparing the cost of each study (calculated by the time of administration × the dose of the drug) we discovered that the cost of this study was significantly lower compared to other studies **(**Table 9). The minimum cost of 6 months was RMB 7324, which was only half of the minimum cost of RMB 15,190 [23] for the study of PAP treatment by inhaling this drug, and only a quarter of the minimum cost of RMB 32,811 Yuan [16] for the study of PAP treatment by subcutaneous injection of this drug. The maximum cost of this study was RMB 13,020, which was 1/26 of the maximum cost of RMB 344,306 [21] for the study of PAP treatment by inhaling this drug and 1/21 of the maximum cost of RMB 284,357 [20] for the study of PAP treatment by subcutaneous injection of this drug.

### Safety analysis

Mild skin stiffness at the subcutaneous injection site (arm) appeared in two patients, and it almost disappeared after 2 weeks of the replacement of the injection site. No other adverse events occurred, and the blood routine, biochemical and other safety indicators were normal.

## Discussion

Whole lung lavage (WLL) treatment has been considered the most effective and preferred treatment approach for pulmonary alveolar proteinosis (PAP). WLL has applied in clinical practice for nearly 50 years [28]. However, some patients show poor or ineffective response to the therapy, or require repeated treatment. Moreover, WLL is an invasive treatment method that needs to be carried out in the operating room under general anesthesia. The risks of anesthesia, washing operation and corresponding economic costs are relatively high. With the deep understanding of the pathogenesis of PAP, subcutaneous injection or inhalation of granulocyte-macrophage colony-stimulating factor (GM-CSF) has become an important treatment technique for idiopathic PAP. Although Cormac McCarthy et al. [29] suggested that statins could be used for PAP treatment by reducing the cholesterol level in alveolar macrophages, the actual application in clinical practice still needs to be confirmed by data of a large number of studies. Therefore, GM-CSF is still considered the main therapeutic drug for PAP.

At present, some studies reported on the treatment of PAP by GM-CSF [15–24]. Of the five studies [15–18, 20] describing the treatment of PAP by GM-CSF subcutaneous injection, a single dose recommended by Seymour [15] was the smallest, which was 5 μg/(kg · day). The study by Seymour included only one patient, whose therapy duration was 300 days and the effective rate was 100%. The single dose of another subcutaneous injection was 9 μg/(kg · day) [16]; a total of four patients were included, and the duration of therapy was 12 weeks. The effective rate of this study was 75%. In another study [18], the single dose of subcutaneous injection was 18 μg/(kg · day), and the duration of therapy ranged from 12 to 48 weeks. Eleven patients were included, and the effective rate was 55%. In another study [20], the single dose of subcutaneous injection was 18 μg/(kg · day), and the duration of medication ranged from 12 to 52 weeks, in which 21 patients were included in the study. In another study [17], the single dose of subcutaneous injection was also 20 μg/ (kg · day). A total of 14 patients were included. The duration of medication was 6–12 weeks, and the effective rate was 43%. Five studies reported on the GM-CSF inhalation treatment for PAP [19, 21–24] of which the minimum single dose of inhalation treatment was 125 μg/kg [24] (twice/day inhalation, 1 week to stop, and therapy time was 24 weeks). A total of 35 patients were included, and the effective rate was 66%. The minimum single dose of another inhalation treatment study [19] was 250 μg/ (kg · day). The medication time was 24 weeks (1 week to stop). Three patients were included, and the effective rate was 100%. In another inhalation treatment study [21], the minimum single dose was 500 μg/kg (twice/day, 1 week to stop), and the duration of medication varied from 3 to 68 weeks. Twelve patients were included, and the effective rate was 92%. In one inhalation treatment study [22], the minimum single dose was 250 μg/(kg · day) (1 week to stop), and the duration of therapy was 24 weeks. Thirty-nine patients were included, and the effective rate was 62%. The minimum single dose of the inhalation treatment study [23] was 250 μg/(kg · day). The duration of therapy was 14–65 weeks, and the treatment was stopped for 4 days after every 4 days. A total of 35 patients were included, and the effective rate was 66%.

In this study, idiopathic PAP was treated by subcutaneous injection of low-dose GM-CSF (75 μg/d or 150 μg/d, ie 1.25 μg/kg/d or 2.5 μg/kg/d, calculated according to 60 kg body weight), which was far lower than the dose reported by other studies. The treatment time was 6 months, and the overall effective rate reached 85%. No obvious adverse reactions were found, and the economic burden of patients was significantly reduced compared with other studies reported so far. This type of therapy is especially suitable for patients with mild disease. In this study, seventeen of the 20 patients were followed up for 6 months after treatment (a total of 12 months), and three of them showed slightly increased lesions after withdrawal, indicating that the disease might relapse after withdrawal. In addition, eight of the 20 patients received a single dose of 75μg for 6 months, and no improvement was observed in 12 patients after 1-month evaluation. The single dose was increased to 150μg from the second month. After the increase of the dose, the efficacy of 9 patients among the 12 patients was evaluated as improved, indicating that appropriate increase of the dose can increase the efficacy. Nevertheless, to what extent can the dose ensure good efficacy, low side effects and the lowest economic burden, these problems need to be confirmed by further research by increasing the sample size.

## Conclusion

Subcutaneous injection of rhGM-CSF at low dose (75μg-150μg /d) is effective treatment for patients with idiopathic PAP.

In conclusion, the method of subcutaneous injection of GM-CSF low dose designed in this study has the advantages of good clinical efficacy, low economic burden and light side effects compared with the methods reported by other studies and is suitable for clinical promotion and application.

## Data Availability

The datasets used and analyzed during the current study are available from the corresponding author on reasonable request.

## Abbreviations

DLCO: Carbon monoxide diffusion capacity
HRCT: High resolution computed tomography
Pa(A-O)2: Alveolar-arterial oxygen partial pressure difference
PaO2: Partial pressure difference
PAP: Pulmonary alveolar proteinosis
PAS: Periodic-acid-schiff
rhGM-CSF: Recombinant human granulocyte-macrophage colony-stimulating factor
WLL: Whole lavage
